# A Microcosmic Syndrome Differentiation Model for Metabolic Syndrome with Multilabel Learning

**DOI:** 10.1155/2020/9081641

**Published:** 2020-11-26

**Authors:** Shujie Xia, Jia Zhang, Guodong Du, Shaozi Li, Chi Teng Vong, Zhaoyang Yang, Jiliang Xin, Long Zhu, Bizhen Gao, Candong Li

**Affiliations:** ^1^Research Base of Traditional Chinese Medicine Syndrome, Fujian University of Traditional Chinese Medicine, Fuzhou 350122, China; ^2^Department of Artificial Intelligence, Xiamen University, Xiamen 361005, China; ^3^State Key Laboratory of Quality Research in Chinese Medicine, Institute of Chinese Medical Sciences, University of Macau, Macao 999078, China

## Abstract

**Background:**

Metabolic syndrome (MS) is a complex multisystem disease. Traditional Chinese medicine (TCM) is effective in preventing and treating MS. Syndrome differentiation is the basis of TCM treatment, which is composed of location and/or nature syndrome elements. At present, there are still some problems for objective and comprehensive syndrome differentiation in MS. This study mainly proposes a solution to two problems. Firstly, TCM syndromes are concurrent, that is, multiple TCM syndromes may develop in the same patient. Secondly, there is a lack of holistic exploration of the relationship between microscopic indexes, and TCM syndromes. In regard to these two problems, multilabel learning (MLL) method in machine learning can be used to solve them, and a microcosmic syndrome differentiation model can also be built innovatively, which can provide a foundation for the establishment of the next model of multidimensional syndrome differentiation in MS.

**Methods:**

The standardization scale of TCM four diagnostic information for MS was designed, which was used to obtain the results of TCM diagnosis. The model of microcosmic syndrome differentiation was constructed based on 39 physicochemical indexes by MLL techniques, called ML-kNN. Firstly, the multilabel learning method was compared with three commonly used single learning algorithms. Then, the results from ML-kNN were compared between physicochemical indexes and TCM information. Finally, the influence of the parameter *k* on the diagnostic model was investigated and the best *k* value was chosen for TCM diagnosis.

**Results:**

A total of 698 cases were collected for the modeling of the microcosmic diagnosis of MS. The comprehensive performance of the ML-kNN model worked obviously better than the others, where the average precision of diagnosis was 71.4%. The results from ML-kNN based on physicochemical indexes were similar to the results based on TCM information. On the other hand, the *k* value had less influence on the prediction results from ML-kNN.

**Conclusions:**

In the present study, the microcosmic syndrome differentiation model of MS with MLL techniques was good at predicting syndrome elements and could be used to solve the diagnosis problems of multiple labels. Besides, it was suggested that there was a complex correlation between TCM syndrome elements and physicochemical indexes, which worth future investigation to promote the development of objective differentiation of MS.

## 1. Introduction

Metabolic syndrome (MS) is a metabolic disorder syndrome, which was characterized by obesity, hyperglycemia, hypertension, dyslipidemia, and hyperuricemia, and this seriously endangers the health of patients [1]. It is a complex multisystem disease, and the difficulty lies between early intervention and multitarget therapy [2]. Traditional Chinese medicine (TCM) has the characteristics of multitarget, small side effects, and regulation [3, 4], so it has attracted more and more attention to the scientists for the prevention and treatment of MS. Many studies have shown that the therapeutic effects of MS in TCM are fairly satisfied [5–7]. Syndrome differentiation is the basis of TCM treatment, which is based on TCM theories and the experiences from the doctors to diagnose patients according to the patient's four diagnosis information of TCM [8]. However, the traditional way of syndrome differentiation inevitably has the problems of subjectivity and fuzziness, which actually prevent the use of TCM [9].

In order to achieve an effective and objective standard for syndrome differentiation, many researchers have investigated the inherent relationship between symptoms and syndromes by using machine learning and data mining methods. For example, to deal with the problems of high nonlinearity and complex interaction of different symptoms [10, 11], many machine learning methods, such as nearest neighbor (kNN), support vector machine (SVM), neural networks (NNs), Bayesian networks (BN), and decision tree (DT), were applied to TCM diagnosis. Specifically, a study introduced the method of SVM for the hypertension diagnosis in TCM, and the experimental results demonstrated that the use of the SVM algorithm to model TCM syndrome diagnosis did not only obtain high accuracy, but also had methodological feasibility [12]. On the other hand, another study used BN for clinical analysis and found that an association mode of symptoms and phlegm-heat congesting lung syndrome was constructed [13]. Given that TCM syndromes can be divided into location and nature syndrome elements [14], various mathematical models were employed to quantify the four diagnostic information [15], thus achieving syndrome element diagnostic task, such as factor analysis and logistic regression analysis for the TCM diagnosis of ulcerative colitis [16].

With the development of modern medicine, many studies have found that TCM syndromes are associated with multidimensional complexity and correlated with multiple microcosmic indexes, and these indexes enable the process of syndrome differentiation to be more objective and standardized [17, 18]. Importantly, microcosmic indexes can be used to assist differentiation syndrome, especially when the symptoms are not discernible. There are some points that can be considered to explore the relationship between different syndromes and physicochemical indexes. For example, the chi-squared automatic interaction detector (CHAID) decision tree was used to build a recognition mode of phlegm-heat stasis syndrome according to the indexes of clinical routine examinations for unstable angina (UA). The results showed that the CHAID decision tree model has certain advantages for the disease recognition [19]. Another study investigated whether these biomedical indexes could be beneficial to TCM syndrome diagnostics for chronic hepatitis B (CHB) patients. It showed that the performance of syndrome classification based on proper integration of TCM and modern clinical indexes was significantly higher than those based on one parameter only. Besides, the correlation analysis and clinical verification test identified potential associations between symptoms and clinical indicators, which are related to mitogen-activated protein kinase (MAPK) signaling pathway [20].

**Figure 1:**
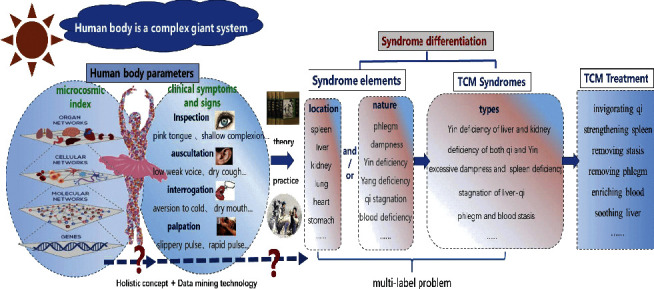
The process of TCM syndrome differentiation.

However, the aforementioned learning methods are aimed at solving the problem of single syndrome diagnosis, i.e., single-label learning, in which this method cannot cope with the problem of multiple syndromes that are occurred at the same time, i.e., multiple labels, simultaneously. In clinical practice, many symptoms are associated with various syndromes [21]. Previous studies [22, 23] have shown that many MS patients always have two or more than two types of syndrome elements. Compared with conventional learning methods, multilabel learning is more capable of identifying syndrome information in TCM, i.e., it can solve the problem of one patient with several syndromes. The research idea of this study is shown in Figure 1. In this study, the TCM information (TI) was obtained to confirm syndrome diagnosis according to syndrome element differentiation, and 39 physicochemical indexes were collected as predictors for diagnostic purposes. Based on the collected physicochemical dataset, the microcosmic syndrome differentiation model for multiple syndromes was constructed by multilabel learning, which has been proved to be feasible and effective [24]. In addition, the influence of the location and the nature syndrome elements on modeling was also assessed.

In Section 2, the dataset, syndromes selection, and multilabel learning methods were described. In Section 3, the results of all the models that were based on ML-kNN, kNN, DT, and SVM were analyzed and the effects of these models were assessed. In Section 4, the reasons why multilabel learning could improve the results were clarified. Finally, we summarized this study in Section 5.

## 2. Materials and Methods

### 2.1. Patients

The inpatients and outpatients with MS were selected in the Second People's Hospital Affiliated to the Fujian University of TCM, the Third People's Hospital Affiliated to the Fujian University of TCM, the Fuzhou Second Hospital, and the Jinjiang Hospital of TCM from 2015 to 2019. All the participants signed the consent forms. Ethics approval for the present study was given by the Medical Ethics Committee of the Fujian University of TCM.

According to the “syndrome differentiation of 600 common symptoms” and the standards of the MS common symptoms in the Guidelines of Clinical Research of TCM New Drugs, the four diagnosis information collection scale was established. The symptoms and signs of disease were classified as none, mild, moderate, and severe with 0, 1, 2, and 3 points, respectively. The four diagnostic data were collected by two qualified professionals of TCM. The physicochemical indexes included the following indexes: body weight, height, blood pressure, abdominal circumference, blood routine, fasting blood sugar, insulin, blood lipid, liver function, and kidney function.

### 2.2. Diagnostic Criteria

The diagnostic criteria of Western medicine: according to the MS diagnostic criteria issued by the International Diabetes Federation (IDF) and American Heart Association (AHA) and Diabetes Society of the Chinese Medical Association, diagnosis can be made if the following 3 or more items are met: (1) abdominal obesity (waist circumference: male ≥ 90 cm and female ≥ 85 cm); (2) hyperglycemia: fasting plasma glucose (FPG) ≥ 6.1 mmol/L or two hours postprandial blood glucose (2 hPG) ≥ 7.8 mmol/L and/or diagnosed as diabetes; (3) hypertension: blood pressure (BP) ≥ 130/85 mmHg and/or diagnosed as hypertension; (4) triglyceride (TG) ≥ 1.70 mmol/L; and (5) high-density lipoprotein cholesterol (HDL-C) < 1.04 mmol/L.

TCM syndrome elements diagnosis standard: according to the Syndrome part of TCM Clinical Diagnosis and Treatment Terminology (the National Standard of the People's Republic of China), syndrome elements differentiation [14] and expert consultation were used.

### 2.3. Inclusion and Exclusion Criteria

The inclusion criteria of the patients are as follows: (1) patients who meet the diagnostic criteria of MS, (2) patients who are given the informed consent, and (3) the ages of the patients ranged from 18 to 75.

The exclusion criteria are as follows: (1) patients with mental diseases or other severe diseases, (2) patients who could not express their feelings clearly, and (3) patients who refuse to participate in our study or without informed consent.

### 2.4. Data Processing

For the selection of syndrome elements, four diagnostic information was input into the TCM syndrome element syndrome differentiation system to obtain the location and nature of syndrome elements, and the results were examined by two professional doctors. Combining with literature reviews, 15 of the most common syndrome elements of MS were selected, where the location syndrome elements include spleen, liver, kidney, lung, heart, and stomach, and the nature syndrome elements include phlegm, dampness, Yin deficiency, Yang deficiency, Qi stagnation, Qi deficiency, blood stasis, and blood deficiency.

For data normalization, dimensionality reduction of the original data was used to eliminate some of the diagnostic information that was never appeared in the dataset. Then, individual missing data were filled, and some cases with too much incomplete data were eliminated. Finally, in order to facilitate data processing and ensure a faster convergence of the running program, the unified normalization of the data was applied.

For computational methods, a model was constructed to map the relationship between microcosmic indexes and syndrome elements by using ML-kNN algorithm. ML-kNN is the extension of the kNN algorithm for multilabel learning, which is used to search for *k* nearest instances with their labels for each test instance, thus predicting the labels of the test instance. kNN is only suitable for processing cases with single labels, whereas the dataset for differentiation diagnosis of MS is suitable for processing cases with multiple labels. For this reason, the multilabel learning algorithm, such as ML-kNN [25], was developed to examine the correlation of the labels. Suppose that there are *p* samples in the test set, the ML-kNN algorithm is shown as follows:

  Step 1: suppose that *t* is an instance in the test set, calculate the distances of the test instance *t* to all the training instances, and find the *k* instances of the training data with the smallest distance
  Step 2: according to the labels of the *k* nearest instances, calculate the number of each label in the *k* nearest instances for test instance *t*
  Step 3: according to the statistical results, predict the posterior probability of *t* on each label
  Step 4: export the posterior probability of all the labels with the naive Bayes method
  Step 5: repeat Steps 1 to 4 until the prediction of all the test instances was finished
  Step 6: assess the predicted results according to multilabel evaluation criteria

### 2.5. Experimental Design and Evaluation

**Figure 2:**
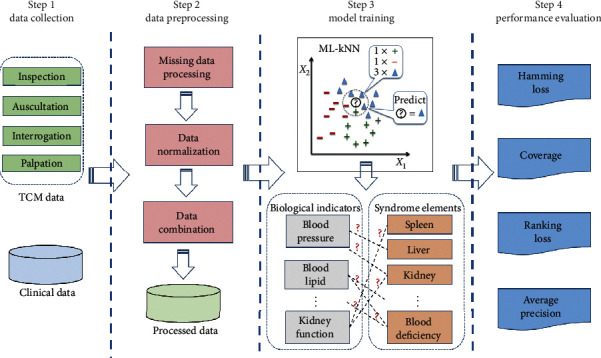
A paradigm of the proposed method.

Specifically, this study used multilabel learning approach to establish the microcosmic syndrome differentiation for metabolic syndromes in TCM. To achieve this purpose, we utilized the collected TCM data and physicochemical data from three TCM hospitals in China. The TCM data were used to get the diagnosis of syndrome elements and physicochemical indexes to train the predictive model. Furthermore, effective data preprocessing methods were also used, including missing data processing, data normalization, and data combination. Then, ML-kNN algorithm was used to explore the potential relationship and rules between physicochemical indexes and syndrome elements, thus establishing a microcosmic syndrome diagnostic model. Finally, four evaluation metrics were used to assess the effectiveness of our proposed method with other state-of-the-art approaches (Figure 2).

In order to ensure the adequacy of the training, 10-fold cross-validation was used for evaluating the performance of this study systematically. Briefly, 90% of the samples were randomly selected as the training set, and the remaining 10% was used as the test set. As the validation was repeated 10 times, the average value was used as the final prediction. All the predicted results from ML-kNN algorithm were used to compare with three other traditional algorithms, namely, KNN, DT, and SVM. It was noted that the evaluation metrics involved in the experiments included Hamming loss (HL), coverage, ranking loss (RL), and average precision (AVP), and the performance of the multilabel algorithm could obtain the objective evaluation data by these metrics [24, 26]. For AVP, the higher the average accuracy is, the better the model is, and for the other metrics, the smaller the evaluation indexes are, the better the model is.

## 3. Results

### 3.1. Basic Information of MS Patients

**Figure 3:**
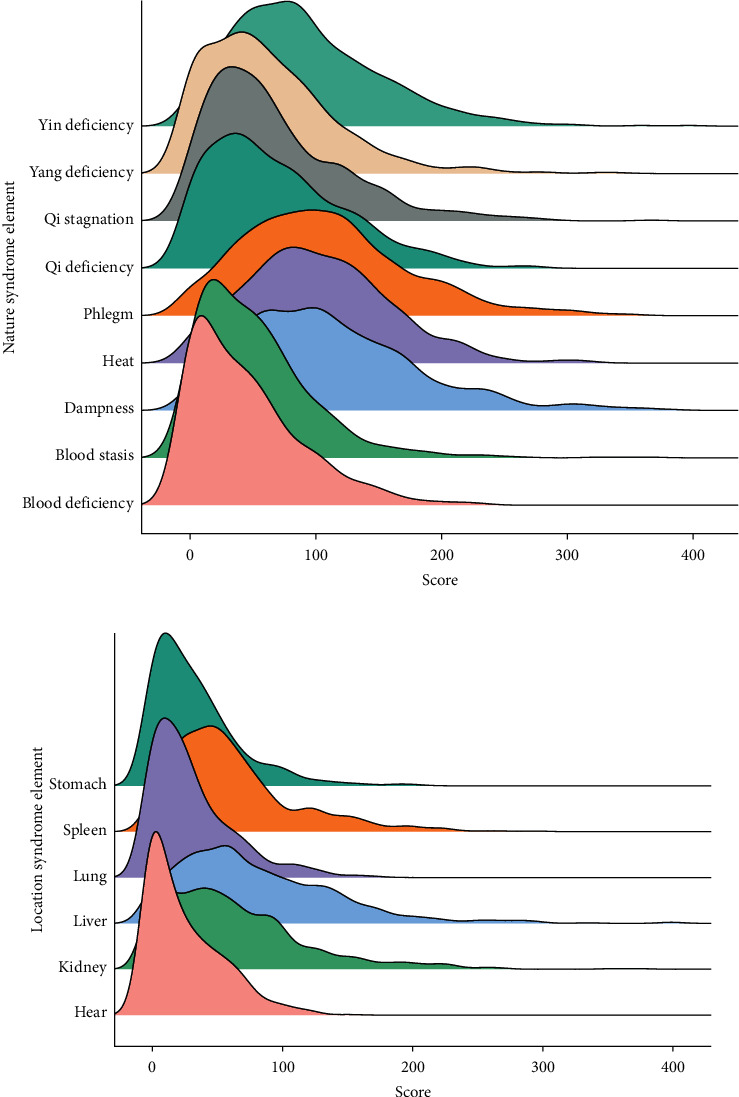
The score distribution of the syndrome elements.

A total of 767 cases were obtained for this study. Among these patients, 396 patients are male (51.6%) and 371 patients are female (48.4%), and the average age of the patients is 44.95 years. After eliminating those patients with too much missing data, 698 patients were selected to conduct this study. Each of them contains four diagnostic information, syndrome identification results, and related microcosmic indexes. The score distribution of the syndrome elements according to syndrome element differentiation is shown in Figure 3, and the basic information of these MS patients is shown in Table 1.

### 3.2. Performance Comparison of Microcosmic Syndrome Models

**Figure 4:**
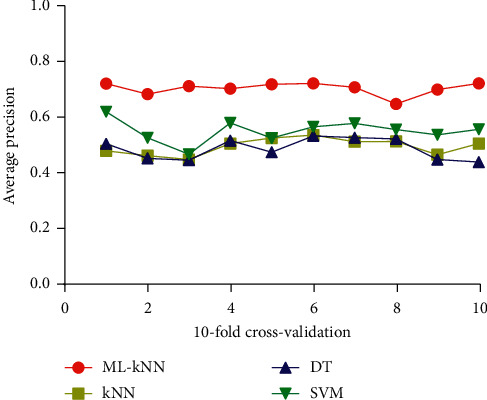
The fluctuation on the average precision of four machine learning in the process of cross-validation.

We used the model of ML-kNN to conduct the experiment in the collected data set, which was set up as described in the Methods section, and the parameter *k* of ML-kNN was set to be 5. In addition, ML-kNN algorithm was compared with kNN, DT, and SVM algorithms. Each prediction model was verified by ten-folds of cross-validation, and the results are shown in Figure 4, where the horizontal coordinate represents the number of cross-validation, and the longitudinal coordinate stands for the average precision and 100% is the highest value. The comparison results of ML-kNN, kNN, DT, and SVM on all the five evaluation metrics are shown in Table 2.

From the results of Figure 4 and Table 2, the following conclusions were made: (1) the average precision of ML-kNN was always higher than kNN, DT, and SVM in the process of ten-folds of cross-validation; (2) for AVP, ML-kNN was 0.714, which was higher than the other methods (kNN, DT, and SVM were 0.217, 0.226, and 0.16, respectively); (3) for HL, ML-kNN was 0.233 and had a lower classification error than kNN, DT, and SVM. Although the differences were small, ML-kNN had the smallest error; (4) for the other evaluation metrics, i.e., RL and coverage, ML-kNN was still ranked higher than kNN, DT, and SVM.

### 3.3. Comparison of Forecast Results between PI and TCM Information (TI)

Generally, the results of TCM syndrome differentiation were obtained through a comprehensive analysis of the TCM information, namely, inspection, auscultation, interrogation, and palpation. In order to further illustrate the diagnostic value of microscopic indexes, a comparison of the prediction results of ML-kNN was applied between PI and TI.

**Figure 5:**
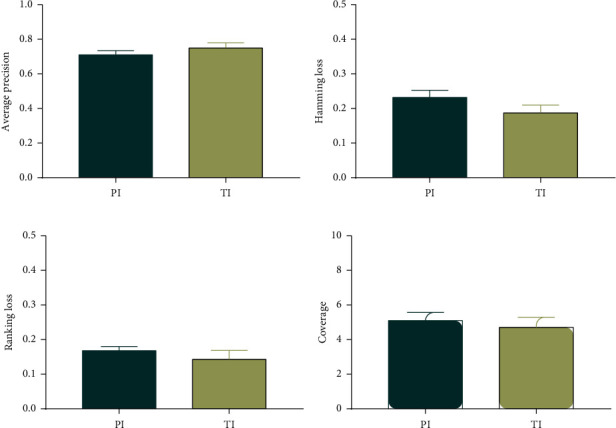
The comparison of the prediction performances of ML-kNN using physicochemical indexes and TCM information.

As shown in Figure 5, the following conclusions were made: (1) Although the prediction results of PI were slightly lower than those of TI, there were no significant differences. (2) For AVP, the values of PI and TI were 0.714 and 0.753, respectively (Figure 5(a)). For HL, the values of PI and TI were 0.233 and 0.188, respectively (Figure 5(b)). For RL, the values of PI and TI were 0.169 and 0.144, respectively (Figure 5(c)). For coverage, the values of PI and TI were 5.123 and 4.731, respectively (Figure 5(d)). Taken together, the results of microcosmic indexes using ML-kNN algorithm were close to that of TCM information.

### 3.4. Effects of Different *k* Values on the Evaluation Metrics

In order to determine whether the *k* value could affect the predicted results of ML-kNN, a model with *k* values of 1, 3, 5, 7, 9, 11, 13, 15, 17, and 19 was constructed. The results are shown in Figure 5. The horizontal coordinate represents the *k* value, and the longitudinal coordinate stands for the average results of AVP, HL, RL, and coverage.

**Figure 6:**
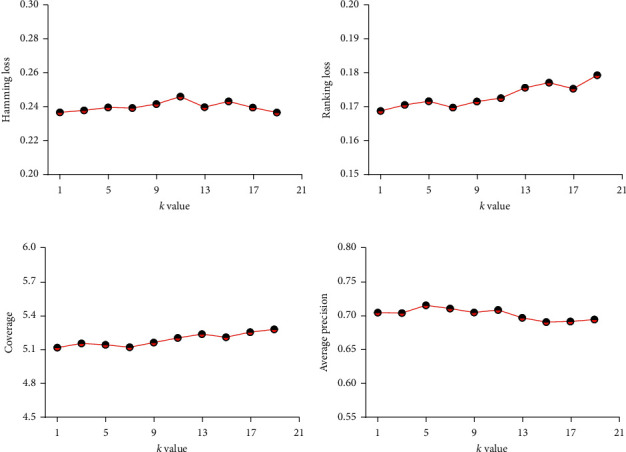
The influence of ML-kNN algorithm using physicochemical indexes on the prediction results with different *k* values.

As shown in Figure 6, the following conclusions were made: (1) the prediction results of ML-kNN have fluctuated with the change of *k* values, but the fluctuation was small; (2) when the *k* value was smaller, AVP was higher, accompanied with lower values of HL, RL, and coverage; (3) in this model, when the *k* value was 5, AVP was higher and the best results of error precision were obtained. When the *k* value was 1 or 3, the result of HL was optimal. When the *k* value was 1 or 7, the best values of RL or coverage were obtained.

### 3.5. Influence of Syndrome Element Selection on Forecast Results

Different types of labels might result in different predicted results. In order to further explore the influence of label selection on the microdiagnostic model of syndrome elements, the average precision of the location and the nature of syndrome elements were analyzed.

**Figure 7:**
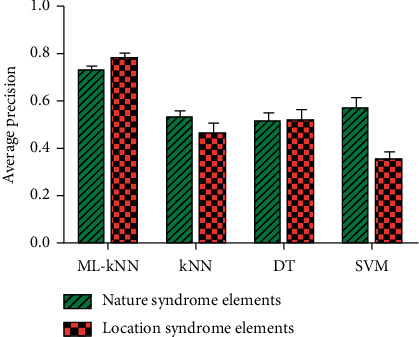
The influence of different syndrome elements on the average precision of ML-kNN using physicochemical indexes.

As shown in Figure 7, the following conclusions were made: (1) The ML-kNN model had the highest prediction values for the location and the nature of syndrome elements, which were 0.728 and 0.776, respectively, while kNN, DT, and SVM had relatively low prediction values. (2) For ML-kNN, the location syndrome elements were better than the nature syndrome elements, while for kNN and SVM, the nature syndrome elements were better than the location syndrome elements. For DT, the differences between location syndrome elements and nature of syndrome elements were not obvious in terms of AVP.

## 4. Discussion

### 4.1. Application of Multilabel Learning Model in TCM Diagnosis

TCM is an empirical medicine, and the discovery of the underlying diagnostic rules can contribute to the development of Chinese medicine. The rules often contain a large number of information on TCM diagnoses and treatment; therefore, the way of how to extract accurate and potential rules from considerable cases becomes an important strategy in the field of TCM. With the introduction of machine learning and data mining technologies, scientists have identified a new way for TCM diagnosis research. TCM syndromes are a complex life system, as it has high-dimensional characteristics [27]. For example, multiple syndrome elements (such as phlegm, Qi deficiency, and spleen) may develop [22]. Besides, there is a primary-secondary relationship between the syndrome elements, so it is unreasonable to use a single element for TCM diagnosis in MS. From the view of computer science, this is a typical multilabel learning problem. In this study, a multilabel algorithm was applied to TCM to establish a diagnostic model, which is suitable for syndrome differentiation. From the training data, the potential correlative rules between multiple TCM information and their syndrome elements and the rules between physicochemical indexes and syndrome elements were obtained. By judging the probability of multiple predicted syndromes, the problem of identification of the primary and secondary syndrome elements at the same time could be solved.

ML-kNN model is a typical multilabel learning algorithm in recent years, which was originally proposed by Zhang and Zhou [25] and was developed based on the traditional single-label kNN model combined with Bayesian algorithm. It has the advantages of simplicity, feasibility, and low error rate [25, 26, 28, 29]. Considering that symptoms and signs or microcosmic indicators do not often appear singly, and the syndrome elements are also related to each other, ML-kNN model can estimate the probability of multiple labels that are related to the final diagnosis as a whole [30], so it is more associated with the core of holistic TCM concept. Our results showed that the prediction results of ML-kNN were better than the traditional single-label algorithms kNN, DT, and SVM. Besides, the *k* value had little influence on the prediction results, indicating that the model is stable and reliable.

### 4.2. Microcosmic Syndrome Differentiation of TCM

Microcosmic syndrome differentiation is the deepening and expansion of traditional syndrome differentiation of TCM. Nowadays, many scientists of TCM syndromes often apply the theory of reduction analysis, and TCM syndromes are attributed to the abnormality in the body. Some scientists are hoping to find a scientific basis or specificity index by using point-to-point research methods. However, in practice, it is difficult to find specific indicators for these syndromes [31]. The reason is that TCM syndromes are of great importance to interrelation and aim to identify the signs of patients from the macrolevel. Its material basis is hard to be interpreted by single or specific indicators. Therefore, in order to explore its diagnostic rules and biological relevance of TCM, we should follow the principles of holism and systematicness and conduct the analysis comprehensively from the relationship and combination of different syndromes.

From this perspective, the multilabel algorithm can be used effectively to explore the relationship between macroscopic syndromes and microcosmic indexes. Our results showed that the average precision of predicting common syndromes of MS could reach 71.4% by using physicochemical indexes. Furthermore, the prediction performance of physicochemical indexes was close to that of TCM information with ML-kNN algorithm, indicating that microcosmic indexes are also helpful for syndrome differentiation of MS. Besides, the AVP of predicting location syndromes could reach 72.8%, while the AVP of predicting nature syndromes could reach 77.6%. This suggested that physicochemical indexes have better performance in predicting the possibility of nature syndrome elements.

In summary, this study promotes the development of microcosmic syndrome differentiation of TCM from a new perspective. As the sample size of this study is limited, future work is warranted to expand the sample size and increase the number of biomedical indexes for microcosmic differentiation. However, the core of TCM syndrome differentiation cannot be separated from macroscopic symptoms, and it is very reasonable to combine microscopic indexes with macroscopic symptoms to determine the most accurate and comprehensive differentiation of TCM syndromes. Taken together, this study provides a research basis for the combination of multiple categories of indicators to improve TCM syndrome differentiation in MS.

## 5. Conclusions

In conclusion, the microcosmic syndrome differentiation model of MS with MLL techniques is good at predicting syndrome elements and can help to solve the diagnosis problem of multiple labels. Besides, we suggested that there is a complex correlation between TCM syndrome elements and physicochemical indexes, and future studies are needed to further promote the development of TCM syndrome differentiation for MS.

## Figures and Tables

**Table 1:** Basic information of MS patients.

Main index	Male (365)		Female (333)		Total (698)	
	Mean	SD	Mean	SD	Mean	SD
Age (years)	42.89	10.85	47.39	11.13	45.04	11.21
BMI	30.80	8.77	30.56	9.72	30.69	9.23
WC (cm)	97.67	6.76	95.46	8.16	96.61	7.54
SBP (mmHg)	133.74	17.28	133.88	19.53	133.81	18.38
DBP (mmHg)	87.70	11.60	84.49	11.72	86.17	11.76
TG (mmol/L)	3.13	3.32	2.36	2.50	2.76	2.98
HDL (mmol/L)	1.20	0.51	1.60	4.33	1.39	3.02
FBG (mmol/L)	10.95	4.75	10.37	4.30	10.67	4.55

WC, waist circumference; SBP, systolic blood pressure; DBP, diastolic blood pressure; TG, triglyceride; HDL, high-density lipoprotein; FBG, fasting blood glucose.

**Table 2:** Evaluation of prediction results from ML-kNN, kNN, DT, and SVM using physicochemical indexes (PI).

Evaluation criteria	ML-kNN	kNN	DT	SVM
Average precision	0.714 ± 0.024^*∗*^	0.497 ± 0.028	0.488 ± 0.036	0.554 ± 0.039
Hamming loss	0.233 ± 0.021^*∗*^	0.297 ± 0.030	0.308 ± 0.020	0.236 ± 0.028
Ranking loss	0.169 ± 0.012^*∗*^	0.698 ± 0.053	0.678 ± 0.044	0.706 ± 0.046
Coverage	5.123 ± 0.476^*∗*^	7.512 ± 0.894	7.866 ± 0.796	7.648 ± 0.743

^*∗*^Representing the index in this model is the best compared with others.

## Data Availability

The data used to support the findings of this study are available from the corresponding author upon request.

## Abbreviations

MS: Metabolic syndrome
TCM: Traditional Chinese medicine
MLL: Multilabel learning
kNN: *k* nearest neighbor
SVM: Support vector machine
NNs: Neural networks
BN: Bayesian networks
DT: Decision tree
UA: Unstable angina
CHB: Chronic hepatitis B
MAPK: Mitogen-activated protein kinase
HL: Hamming loss
RL: Ranking loss
AVP: Average precision
PI: Physicochemical index
TI: TCM information.
